# Clinical value of vestibulo-ocular reflex in the differentiation of spinocerebellar ataxias

**DOI:** 10.1038/s41598-023-41924-6

**Published:** 2023-09-07

**Authors:** Jae-Myung Kim, Tai-Seung Nam, Seong-Min Choi, Byeong C. Kim, Seung-Han Lee

**Affiliations:** Department of Neurology, Chonnam National University Hospital, Chonnam National University Medical School, 42 Jebong-ro, Dong-gu, Gwangju, 61469 Korea

**Keywords:** Neurodegenerative diseases, Spinocerebellar ataxia

## Abstract

The diagnosis of spinocerebellar ataxia (SCA) currently depends upon genetic testing. Although genetic testing for SCA is highly specific, clinical parameters for the differentiation of SCAs are still insufficient. We aimed to assess the vestibulo-ocular reflex (VOR) parameters of various SCA subtypes to determine whether they have substantial value in differential diagnosis. We consecutively enrolled 33 genetically confirmed SCA patients (SCA2 = 8, SCA3 = 6, SCA6 = 10, SCA7 = 9). Normative data were obtained from 36 age- and gender-matched healthy controls. Quantitative indicators of VOR were measured using video head impulse test (HIT) and combined ocular motor dysfunctions were investigated using video-oculography. Compared with the control group, the VOR gains in SCA2 were relatively spared, but were markedly decreased for all six canals in SCA3. The VOR gains for the posterior canals (PCs) were significantly decreased in SCA6, and for both vertical canals were decreased in SCA7. The VOR gains for the horizontal canals in SCA3 were negatively correlated with disease severity (R = -0.900, *p* = 0.037). Abnormal catch-up saccades were common in SCA3 and SCA6, rare in SCA7 and absent in SCA2. Spontaneous, headshaking-induced, and positional nystagmus were only documented in SCA6. SCA3 and SCA6 commonly showed horizontal gaze-evoked nystagmus, but SCA2 and SCA7 had characteristic saccadic slowing without gaze-evoked nystagmus. VOR impairments are common in SCAs, but their patterns vary depending on subtype. In addition to ocular motor characteristics, distinctive VOR performance for each subtype using video HIT may aid the differential diagnosis of the SCA genotypes.

## Introduction

Spinocerebellar ataxia (SCA) is a heterogeneous group of autosomal dominantly inherited, neurological disorders characterized by progressive cerebellar ataxia associated with neurodegeneration of the cerebellum and its connections^1–3^. SCA may also exhibit various additional symptoms including pyramidal/extrapyramidal signs and ophthalmoplegia as well as all the classic cerebellar dysfunction signs^4^. However, since there is considerable overlap between the clinical phenotypes of SCA, the differential diagnosis of SCA is usually dependent upon the genetic testing^1,2,4^. Although genetic testing for SCA is highly specific and can reliably identify the genotype, it is still limited in clinical practice^1,2,4^. Characteristic clinical features may be unapparent in earlier stages of the disease. Therefore, clinicians are often hesitant to test for all the different genetic subtypes in a suspected case since it is a quite expensive and time-consuming process. From a diagnostic point of view, identifying and developing clinical differentiating factors will be critical for narrowing the differential diagnosis before the particular subtype is confirmed by molecular diagnosis.

Previous studies have shown that vestibulo-ocular reflex (VOR) is impaired in several types of SCA and that the detection of VOR deficits could be a useful clinical indicator^5–8^. However, the results of the previous studies regarding the VOR in SCA were inconsistent according to the accelerations of the stimuli and measurement techniques^6,8–13^. Moreover, there have been few comparative studies among various SCA subtypes, particularly including the SCA7^8,14^. Therefore, this study aimed to quantitatively assess VOR function in SCA patients with four representative subtypes (SCA2, SCA3, SCA6, and SCA7) using a video head impulse test (HIT) device, which particularly measure VOR with high frequency, to determine whether it has substantial value in the differential diagnosis.

## Patients and methods

### Study population and clinical evaluation

**Figure 1:**
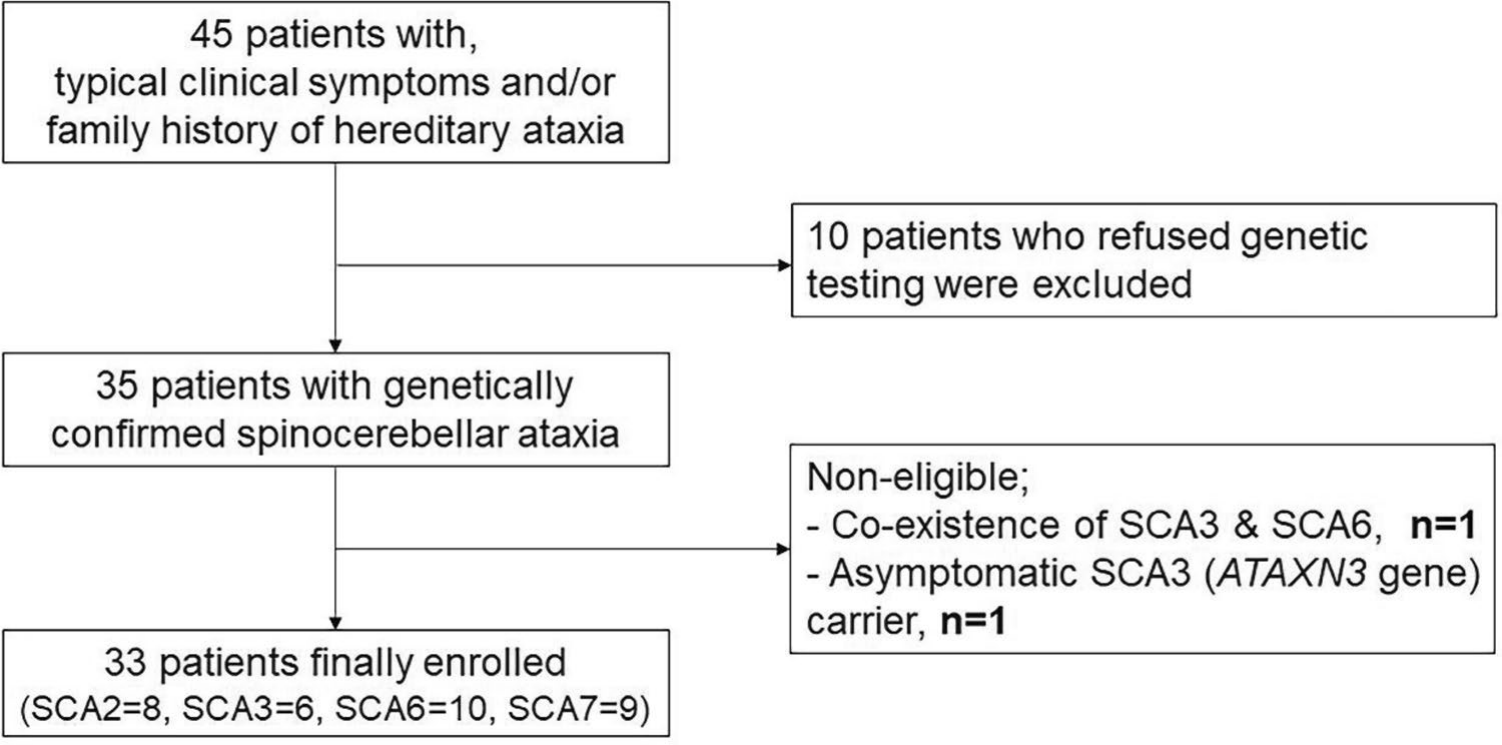
Flow diagram of the study.

Of the patients with typical clinical symptoms and/or family history of cerebellar ataxia who visited the Chonnam National University Hospital between January 2015 and August 2022, 45 were eligible for the study. A diagnosis of SCA was made by: (1) typical, progressive cerebellar symptoms such as dysarthria, limb and truncal ataxia; (2) genetic confirmation as a pathological expansion of CAG trinucleotide repeat on novel genes (i.e., SCA2, *ATXN2*; SCA3, *ATXN3*; SCA6, *CACNA1A*; SCA7, *ATXN7*). Expanded CAG repeats defined as follows: SCA2 ≥ 33, SCA3 ≥ 52, SCA6 ≥ 20, and SCA7 ≥ 38^1^. Among 35 genetically confirmed patients, two patients were excluded; one patient who has co-mutations of SCA3 and SCA6, and one SCA3 patient who was asymptomatic *ATAXN3* gene carrier. A total of 33 patients (men = 17, mean age ± SD = 45.9 ± 14.5 years, SCA2 = 8, SCA3 = 6, SCA6 = 10, SCA7 = 9) were finally enrolled in the study (Fig. 1). For normative data, a group of 36 age- and gender-matched healthy volunteers (men = 18, mean age ± SD = 42.4 ± 15.5 years) with no family history of SCA or any history of vestibular, cochlear, and ocular motor disorders were recruited as controls.

Baseline demographics including the age at onset of initial symptoms, disease duration, and genetic information (i.e., CAG repeat length) were investigated. To assess disease severity, the Scale for the Assessment and Rating of Ataxia (SARA) along with detailed bedside neuro-otologic and neuro-ophthalmologic examinations were performed^15,16^.

MRI was performed in all patients. Cerebellar atrophy was considered when some cerebellar sulci were visible either in the mid-sagittal or axial T1-weighted images. FLAIR images were used when T1-weighted images were unavailable. Brainstem atrophy was measured by visually evaluating the width of the prepontine cistern, the shape of the basis pontis, and the size of the fourth ventricle^17^.

### Video head impulse testing

All patients and healthy controls underwent quantitative head impulse testing using a lightweight, portable video HIT device (ICS Impulse; GN Otometrics, Taastrup, Denmark). Detailed testing methods have been described previously^18^. Abnormal VOR gain was defined when the estimated gain value deviated from the mean ± 2SD obtained from the control group [normal gains for the horizontal canals (HCs) = 0.8–1.24, for the anterior canals (ACs) = 0.73–1.13, and the posterior canals (PCs) = 0.73–1.08]. The mean VOR gain during 20 valid trials for HCs, ACs, and PCs on both sides was adopted for statistical analysis.

Bedside HITs for the HCs were performed with all patients to detect perverted HIT which was defined as showing a dynamic upward bias, so that the eyes moved upward as well as horizontally, producing a “cross-coupled” VOR during the horizontal head impulses^19^.

### Eye movement recordings

All patients had three-dimensional video-oculography (VOG; SLMed, Seoul, Korea) in a sitting position to assess ocular motility. Spontaneous nystagmus (SN) and provoked nystagmus [i.e., headshaking-induced nystagmus (HSN), gaze-evoked nystagmus (GEN), and positional nystagmus) were recorded binocularly. Perverted HSN was defined as vertical and/or torsional nystagmus in response to horizontal head shaking^20,21^. GEN was only considered to be present when the recorded nystagmus beat in the direction of gaze bilaterally^22^. To induce positional nystagmus, positional testing including the supine roll test and the Dix-Hallpike maneuver was performed in a sequential manner^23^. Horizontal and vertical saccades and smooth pursuits were also investigated using the same VOG instruments. Detailed testing methods also have been described previously^24^.

### Statistical analyses

Statistical analyses included intergroup comparisons of the Chi-square or Fisher’s exact test for dichotomous variables. Additionally, the Mann–Whitney U-test, a one-way analysis of variance (ANOVA) and the Kruskal–Wallis test, followed by post-hoc pairwise comparisons using Mann–Whitney U-tests (one-tailed, Bonferroni corrected for multiple comparisons) were all used for continuous variables. Correlation analyses between two variables were performed using Spearman’s correlation. All tests were performed using SPSS version 26.0 (SPSS Inc, Chicago, IL, USA), with *p* values of < 0.05 denoting statistical significance.

### Ethics approval

This study was performed in accordance with the recommendations of the Institutional Review Board of Chonnam National University Hospital (Gwangju, South Korea). The authors declare that they acted in accordance with the ethical standards laid down in the 1964 Declaration of Helsinki.

### Consent to participate

Informed consent was obtained from all individual participants included in the study.

## Results

### Baseline characteristics and neuroimaging findings

The baseline demographics and clinical characteristics of each SCA patient group are summarized in Table 1 (more detailed, individual information are shown in Supplementary Table S1). Except for CAG repeat length, the clinical characteristics of the study population (i.e., mean age, disease duration, gender ratio, SARA, and age at onset) did not differ among each subtype (Table 1). The most common initial symptom of SCA patients was gait ataxia (45.5%), followed by intermittent imbalance (36.4%), and dysarthria (21.2%). There was no difference in the initial symptoms according to the subtype. However, among six patients (18.2%) who complained of dizziness/vertigo as an initial symptom, four of them were diagnosed with SCA6. All of the SCA patients exhibited cerebellar atrophy with or without brainstem atrophy (Supplementary Fig. S1). Brainstem atrophy was also observed in 22 SCA patients (66.7%) although it was rare in the SCA6 subgroup (10% vs. SCA2, 100%; SCA3, 66.7%; SCA7, 100%).

**Table 1:** Clinical characteristics and video head impulse test results among the control group and each subgroup of spinocerebellar ataxia.

	Control (n = 36)	SCA2 (n = 8)	SCA3 (n = 6)	SCA6 (n = 10)	SCA7 (n = 9)	*p*-value
Clinical characteristics						
Age (year, mean ± SD)	42.4 ± 15.5	40.9 ± 13.2	50.0 ± 11.6	55.3 ± 11.4	37.2 ± 14.9	0.064
Male (n, %)	18 (50.0%)	5 (62.5%)	3 (50.0%)	5 (50.0%)	4 (44.4%)	0.946
Age at onset (year, mean ± SD)	N/A	31.4 ± 13.3	41.8 ± 15.4	40.9 ± 8.7	29.9 ± 15.4	0.178
Disease duration* (year, mean ± SD)	N/A	6.1 ± 6.2	6.5 ± 4.8	10.3 ± 8.0	5.4 ± 2.9	0.265
CAG repeat length ^†^ (mean ± SD)	N/A	41.9 ± 2.6 (37–45)	71.0 ± 4.7 (66–79)	25.1 ± 2.2 (21–27)	46.9 ± 6.7 (40–57)	
SARA^‡^ (mean ± SD)	N/A	11.3 ± 4.8	14.4 ± 4.6	18.3 ± 10.4	14.8 ± 6.8	0.371
VOR parameters						
VOR gains, median (IQR)						
HC	1.03 (0.93–1.07)	1.03 (0.96–1.10)	↓0.63 (0.55–0.76)^§^	0.95 (0.77–1.24)	0.94 (0.73–1.06)	0.002
AC	0.92 (0.87–1.01)	0.95 (0.88–0.99)	↓0.62 (0.52–0.70)^§^	0.87 (0.76–0.95)	↓0.54 (0.44–0.73)^§^	< 0.001
PC	0.91 (0.84–0.96)	0.89 (0.77–1.08)	↓0.59 (0.55–0.82)^§^	↓0.68 (0.57–0.83)^§^	↓0.78 (0.59–0.88)^§^	< 0.001
AC/PC	0.93 (0.82–1.01)	1.02 (0.91–1.21)	0.92 (0.83–1.06)	↑1.19 (1.14–1.51)^§^	↓0.79 (0.66–0.85)^§^	< 0.001
*Catch-up saccades*						
Overt saccades	0	0	5 (83.3%)	8 (80%)	1 (11.1%)	
Covert saccades	0	0	4 (66.7%)	4 (40%)	0	

*Disease duration was calculated from the age at onset to the age when the video head impulse test was performed, ^†^CAG repeat length of SCA6 was available in 9 patients, ^‡^SARA was checked in 26 patients (SCA2 = 7, SCA3 = 5, SCA6 = 7, SCA7 = 7) at the time of video head-impulse test, ^§^Statistically significant when compared with the control group (*p* < 0.001 except for the AC/PC of the SCA7 with *p* = 0.001). VOR:  vestibulo-ocular reflex, IQR: interquartile range, HC:  horizontal canal, AC:  anterior canal, PC:  posterior canal, SARA: Scale for the Assessment and Rating of Ataxia, SCA: spinocerebellar ataxia.

### Video HIT findings

#### VOR gains

**Figure 2:**
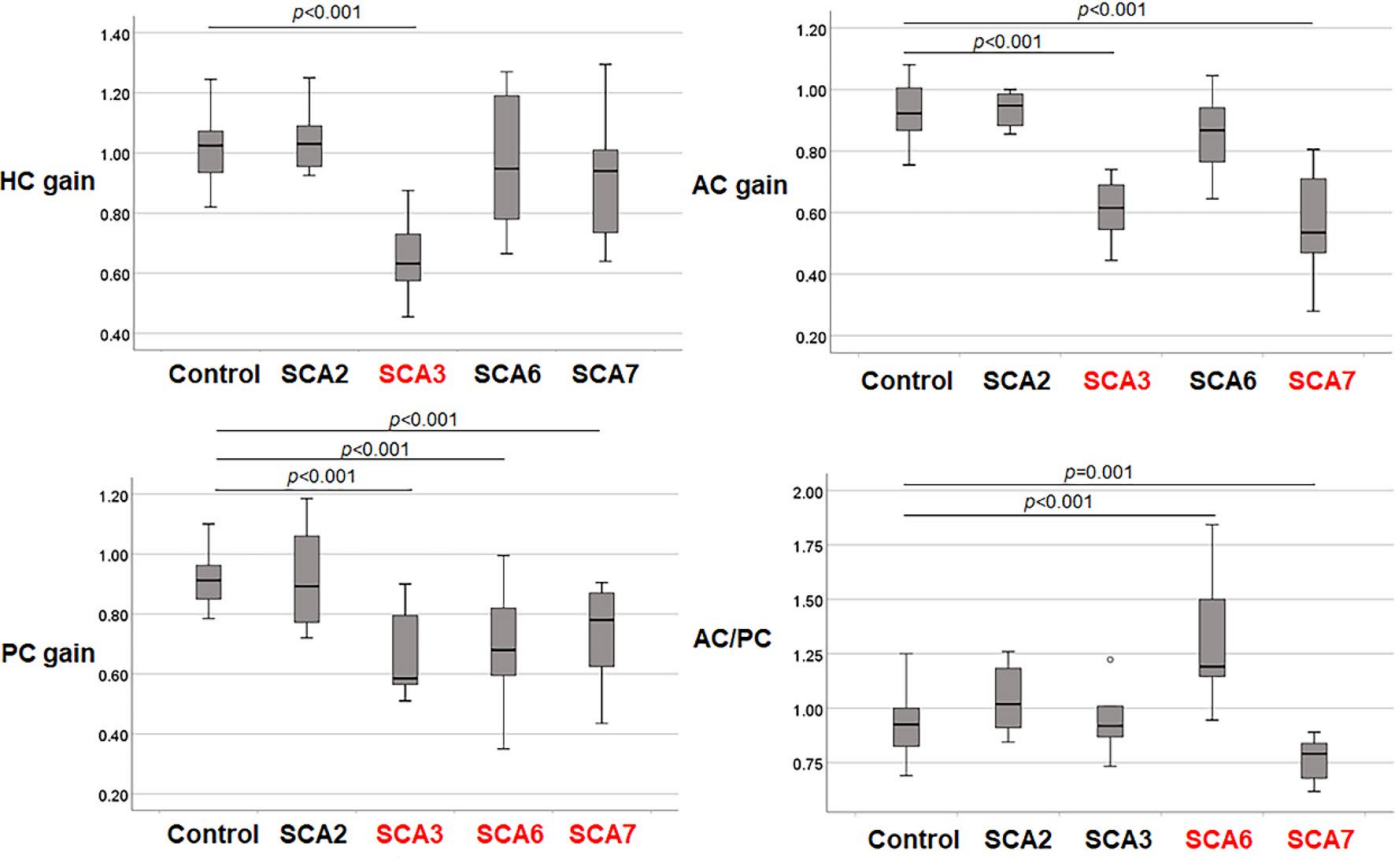
Distribution of the vestibulo-ocular reflex (VOR) gains according to the canal plane.

Among the 33 enrolled patients with SCA in whom VOR gains were measured by the video HIT device, 28 (84.8%; SCA2 = 5, SCA3 = 6, SCA6 = 8, SCA7 = 9) showed abnormal VOR gains in at least one semicircular canal (Supplementary Table S2). When compared with the control group, the VOR gains of the SCA2 patients tended to be spared (Table 1, Fig. 2A–C). The VOR gains of the SCA3 patients were markedly decreased for both the horizontal and vertical canals [SCA3 vs. control; HCs, 0.63 (median) vs. 1.03, *p* < 0.001; ACs, 0.62 vs. 0.93,* p* < 0.001; PCs, 0.59 vs. 0.91,* p* < 0.001]. In the SCA6 patients, the VOR gains for the PCs were significantly decreased (0.68; *p* < 0.001) and were associated with relatively preserved gains for the ACs. By contrast, the VOR gains of both vertical canals were decreased in the SCA7 patients (ACs, 0.54, *p* < 0.001; PCs, 0.78, *p* < 0.001). For further analysis of the asymmetry of the VOR gains for the vertical canals, we adopted the AC/PC ratio of the VOR gains for each subtype (Table 1, Fig. 2D). Compared with the control group, the SCA6 subgroup showed an increased AC/PC ratio (SCA6, 1.19 vs. the control*,* 0.93; *p* < 0.001), which suggested a marked decrease in the PCs compared with the ACs. In contrast, the SCA7 subgroup consistently exhibited a decreased AC/PC ratio (0.79; *p* = 0.001). The video HIT results of representative cases for each subtype of SCA are shown in Supplementary Fig. S2A–S2D.

Compared with the control group, (A) the VOR gains for the horizontal canal decreased significantly in the SCA3 patients (*p* < 0.001). (B) The VOR gains for the anterior canal (AC) are decreased in the SCA3 (*p* < 0.001) and SCA7 patients (*p* < 0.001), (C) while the VOR gains for the posterior canal (PC) are decreased in SCA3 (*p* < 0.001), SCA6 (*p* < 0.001), and SCA7 (*p* < 0.001). (D) The AC/PC ratio, which is adopted to compare the asymmetrical impairments on the vertical VOR gains among the SCA subtypes, shows a significant increase in the SCA6 patients (*p* < 0.001) and a decrease in the SCA7 patients (*p* = 0.001).

### Analysis of catch-up saccades

Abnormal catch-up saccades during video HITs were documented in 14 patients (42.4%; SCA2 = 0, SCA3 = 5, SCA6 = 8, SCA7 = 1) (Table 1). Among them, eight patients (8/14, 57.1%; SCA3 = 4, SCA6 = 4) also showed covert saccades. Catch-up saccades were frequently observed in SCA3 (83.3%) and SCA6 patients (80%), but catch-up saccades in the PCs were only observed in SCA6 patients. In contrast, catch-up saccades were rare in SCA7 (11.1%) and absent in SCA2 patients. Perverted HITs were documented in four patients (4/13, 30.8%; SCA3 = 1, SCA6 = 3).

For the canal plane, catch-up saccades in the HCs were not associated with decreased VOR gains for the HCs. In detail, four patients (SCA3 = 1, SCA6 = 3) with catch-up saccades in the HCs showed normal VOR gains for the HCs, while three patients (SCA3 = 1, SCA7 = 2) with no catch-up saccades showed decreased VOR gains for the HCs. By contrast, in the SCA6 subgroup, the VOR gains for the PCs with catch-up saccades in the PCs were significantly lower than the VOR gains with no catch-up saccades [median = 0.55 (IQR = 0.39–0.63) vs. 0.8 (0.70–0.90), *p* = 0.011].

### Correlation of HITs with clinical parameters

Correlations between the VOR gains from video HITs and clinical parameters (i.e., CAG repeat length, age at onset, and SARA) varied according to the SCA subtypes (Supplementary Table S3). In the SCA3 subgroup, the VOR gains for the HC were correlated negatively with the SARA (R = − 0.900, *p* = 0.037). The VOR gains did not correlate with clinical parameters in the other SCA subgroups. Except SCA2, age at onset showed an inverse correlation with CAG repeat length in the other SCA subgroups.

### Ocular motor characteristics

The patients’ ocular motor characteristics are summarized in Table 2. Fixation abnormality such as square-wave jerk and macrosaccadic oscillation was observed in four patients (12.1%; SCA2 = 1, SCA3 = 3). SN or provoked nystagmus (HSN and positional nystagmus), except for GEN, was only documented in the SCA6 subgroup (80% vs. 0% in the other three types, *p* < 0.001). SN was documented in seven patients (70.0%) with the direction mostly downbeat (4/7, 57.1%) albeit with occasional horizontal components. Positional nystagmus was documented in seven patients (7/10, 70.0%) and it was mostly apogeotropic direction-changing horizontal nystagmus (85.7%) or geotropic nystagmus (14.3%) during the supine head roll test. Similarly, HSN was shown in eight patients (8/10, 80.0%), with the majority (75.0%) demonstrating perverted response. Of note, horizontal GEN was frequently observed in the SCA3 (83.3%) and SCA6 (90.0%) subgroups, while it was absent in the SCA2 and SCA7 subgroups.

**Table 2:** Ocular motor characteristics of the patients with spinocerebellar ataxia.

	SCA2 (n = 8)	SCA3 (n = 6)	SCA6 (n = 10)	SCA7 (n = 9)
*Fixation abnormality*	1 (12.5%, SWJ)	3 (50.0%, MSO 1, SWJ 2)	0	0
*Spontaneous nystagmus*	0	0	7 (70%)*	0
*Gaze-evoked nystagmus*	0	5 (83.3%)	9 (90%)	0
*Headshaking-induced nystagmus*	0	0	8 (80%, perverted 6)	0
*Positional nystagmus*	0	0	7 (70%)^†^	0
*Saccadic impairment*				
Slowing	7 (87.5%)	2 (33.3%)	0	8 (88.9%)
Hypometria	4 (50.0%)	3 (50.0%)	7 (70%)	3 (33.3%)
Hypermetria	0	2 (33.3%)	1 (10%)	3 (33.3%)
*Smooth pursuit impairment*				
Decreased gain	5 (62.5%)	6 (100%)	6 (60%)	4 (44.4%)
Saccadic pursuit	8 (100%)	6 (100%)	10 (100%)	9 (100%)

SCA: spinocerebellar ataxia, HIT: head impulse test, SWJ: square-wave jerk, MSO: macrosaccadic oscillation.

*The direction of spontaneous nystagmus was either downbeat (4/7, 57.1%) occasionally with horizontal components, or horizontal-beating (3/7, 42.9%). ^†^The direction of positional nystagmus was mostly apogeotropic direction-changing horizontal nystagmus (6/7, 85.7%) during the supine head roll test (1 geotropic nystagmus, 14.3%).

Except for three patients (SCA2 = 1, SCA6 = 2), abnormal saccades (i.e., saccadic dysmetria, saccadic slowing) were observed in all SCA subtypes (90.9%). More specifically, saccadic dysmetria was documented in the form of either hypometria (17/23, 73.9%) or hypermetria (6/23, 26.1%). Additionally, approximately half of the SCA patients (51.5%) exhibited saccadic slowing. Notably, saccadic slowing was common in the SCA2 (87.5%) and SCA7 (88.9%) subgroups, but was absent in the SCA6 subgroup. Saccadic smooth pursuit, either with (63.6%) or without (36.4%) decreased smooth pursuit gain, was invariably documented in all SCA patients.

## Discussion

### Vestibular performance

#### SCA2

Except for one patient who demonstrated mildly impaired VOR gains for the vertical canal, the VOR gains in the SCA2 patients were mostly within the normal range or were mildly increased. Abnormal catch-up saccades during video HITs were also absent. These findings were consistent with previous studies showing normal to minimal impairment of the VOR in SCA2 (Table 3)^8,14,25^. In detail, previous studies showed normal VOR gains in SCA2 during video HITs as well as low-to-intermediate frequency VOR tests (e.g., rotatory chair test), accompanied by infrequent abnormal catch-up saccades^8,25^. Since cerebellum and brainstem neurodegeneration were common in the SCA2 patients, the preservation of the VOR gains could be a peculiar characteristic of SCA2 different from other subtypes. Based on the ocular motor characteristics of SCA2 patients (i.e., frequent saccadic slowing but a paucity of SN and provoked nystagmus), saccadic generator dysfunction may be predominant, whereas main structures for central VOR pathway such as the medial vestibular nucleus (MVN) in the brainstem or the flocculus/paraflocculus in the cerebellum may be relatively spared than in other subtypes of SCA.

**Table 3:** Summary of previous studies regarding the vestibulo-ocular reflex in spinocerebellar ataxia.

SCA types	Patient No	Methods (stimulation)	Major findings	Ref
SCA2	4	Video HIT	Normal VOR gain & latency (100%)	− 8
	14	Rotatory chair test, caloric test	Decreased VOR gain in 30% (not statistically significant), reduced caloric response (75%)	− 14
	5	Rotatory chair test	Normal VOR gain (100%), mildly impaired VOR fixation suppression gain	− 25
SCA3	7	Caloric test, bedside HIT	Absent caloric response (85.7%), abnormal bilateral CS (7/7, 100%), no correlation between the VOR loss and the cerebellar impairment	− 7
	15	Video HIT	Decreased VOR gain (87%), abnormal CS, negative correlation between the VOR gain and the SARA	− 8
	10	MSC HIT, Bedside HIT	Decreased VOR gain (80%), abnormal CS (80%), abnormal CSs were correlated with decreased VOR gain	− 13
	20	Rotatory chair test, caloric test	Decreased VOR gain (70%), reduced caloric response (57.9%), negative correlation between the VOR gain and the CAG repeat length	− 14
	7	Rotatory chair test	Decreased VOR gain (28.6%), mildly impaired VOR fixation suppression gain	− 25
	2	Caloric test	Absent caloric response (100%)	− 26
	21	Video HIT*	Decreased VOR gains for both horizontal and vertical canals (HIMP test), decreased VOR gains for horizontal canals (SHIMP test)	− 27
	6	Rotatory chair test, MSC HIT	Abnormal VOR gain during HIT (100%), abnormal visually enhanced VOR gain (83.3%), normal response to the VOR cancellation (100%)	− 28
SCA6	11	Rotatory chair test, caloric test MSC HIT*	Rotatory chair/caloric test: normal or increased regardless of the severity of disease HIT: VOR gains were increased in the mild cases and decreased in the severe cases, VOR gains during HIT were negatively correlated with the severity of disease (ICARS)	− 6
	12	Video HIT*	HC gain: decreased (33.3%) or increased (16.7%), AC gain: increased (33.3%), PC gain: decreased (33.3%) or increased (16.7%); AC gains > PC gains	− 12
			Follow-up tests: HC and AC gains were decreased, but PC gains remained unchanged	
			Perverted responses during HIT (58%)	
			VOR gains for each canal showed a negative correlation with the SARA	
	5	Rotatory chair test	Normal VOR gain (100%), severely impaired VOR fixation suppression gain	− 25
SCA7	2	Rotatory chair test	Normal VOR gain (100%), mildly impaired VOR fixation suppression gain	− 10

SCA: spinocerebellar ataxia, HIT: head impulse test, CS: catch-up saccade, SARA: Scale for the Assessment and Rating of Ataxia, VEMP:  vestibular evoked myogenic potential, HIMP: head impulse test protocol, SHIMP:  suppression head impulse test protocol, ICARS: International Cooperative Ataxia Rating Scale, HC:  horizontal canal, AC: anterior canal, PC: posterior canal, MSC:  magnetic search coil.

*These studies measured VOR gains for vertical canals as well as horizontal canals. In the other studies, only the VOR gains for horizontal canals were evaluated.

### SCA3

Several previous studies showed decreased VOR gains with abnormal catch-up saccades in SCA3 regardless of the stimulation frequency, though the evaluation of the VOR gains for the vertical canals was scant (Table 3)^7,8,13,25–28^. Our study revealed markedly decreased VOR gains during video HITs regardless of canal plane, associated with frequent abnormal catch-up saccades, which were also in line with previous studies^8,27^. Based on previous neuropathological studies, the mechanisms of the VOR dysfunction in SCA3 may be ascribed to the neurodegeneration of both the MVN and the nucleus prepositus hypoglossi or the bilateral involvement of primary vestibular neurons^13,25^.

### SCA6

Previous studies concerning vestibular performance in SCA6 showed asymmetrical VOR damage, which was more prevalent in the PCs than the ACs during the video HITs (Table 3)^6,12^. By contrast, the vestibular responses to low-frequency stimuli tended to be spared regardless of disease severity^6,12^, suggesting that the discrepancy might be associated with the neurodegeneration of the flocculus or vestibular nuclei^6^. Experimental studies showed that the reduced VOR gains with high-frequency stimuli might be ascribed to a frequency-dependent VOR enhancement of the flocculus^6,12,29^. Indeed, a previous study showed bilaterally decreased VOR gains during HITs (high-frequency stimuli) in the isolated floccular lesion, while the bithermal caloric tests (low-frequency stimuli) were normal^30^. Inhibitory projection of Purkinje cells of the flocculus and ventral paraflocculus to a subset of secondary vestibular nuclei is predominant for the AC and HC pathways^29^. Therefore, losing the inhibitory projection of the flocculus to the AC pathway may result in higher gains for downward impulses than those for upward impulses, as well as perverted HIT/HSN^6,12,29^. In this study, SCA6 patients showed a significant decrease of the VOR gains for the PCs than the ACs along with frequent GEN, spontaneous/positional downbeat nystagmus, and perverted HIT/HSN, which were all common in lesions involving the flocculus.

### SCA7

Vestibular performance in SCA7 has rarely been studied (Table 3)^10,31^. A previous study that assessed VOR using the rotatory chair test showed both low pursuit gains and impaired VOR fixation but showed normal VOR gains in one proband of SCA7 and her mother with the same genetic mutation^10^. Our study revealed that when measured by video HITs, the VOR gains in the SCA7 patients were mostly impaired, particularly for the vertical canals (77.8%).

Interestingly, the impairment of the VOR gains in SCA7 was greater for the AC than the PC (i.e., decreased AC/PC ratio). Although the pathomechanism of the AC-dominant VOR impairments in SCA7 is still to be elucidated, neurodegeneration involving the brachium conjunctivum or the ventral tegmental tract, which consists of the extra-medial longitudinal fasciculus pathway that conveys the VOR signal from the AC, may be responsible^5,32^. Otherwise, neurodegeneration of the superior vestibular nucleus (SVN) which receives excitatory inputs from the AC but not from the PC might also contribute to our results, although neuropathological studies to prove our theoretical assumptions are warranted^5^.

An earlier study that investigated the vestibular function in 16 patients with retinitis pigmentosa (RP) showed frequent vestibular dysfunction (81.3%), even without persistent vestibular disorders^33^. From that study, eight patients with RP (50%) exhibited abnormal responses in at least one semicircular canal^33^. More specifically, the impairment of the VOR gains was more frequent for the ACs (AC = 8, HC = 2, PC = 2)^33^. The study’s authors suggested that these discrete and latent vestibular impairments might favor progressive vestibular sensory cell alteration^33^. Indeed, one of the major characteristic features of SCA7 is pigmentary macular degeneration, which results in color vision and visual acuity abnormalities^1,3,34^. Additionally, all of the SCA7 patients in our study had pigmentary macular degeneration with visual impairment and demonstrated similar patterns of VOR injury to the previously reported study (i.e., predominant impairment for the ACs)^33^. However, since RP is a heterogeneous group of inherited disorder, and the additional information on symptoms and diagnosis from the study patients was lacking, interpretation of vestibular dysfunction in RP requires attention^33^. Although a previous study reported that visual acuity itself might not influence video HIT outcomes including VOR gain^35^, we cannot currently exclude the influence of the RP on the VOR impairments in the SCA7 patients and further investigation is needed.

### Catch-up saccades

Catch-up saccades during HITs are considered to be compensatory saccadic eye movements that usually suggest unilateral peripheral vestibular loss^36^. However, various neurological disorders involving the central vestibular pathway may also show catch-up saccades during HITs^36^. Along with overt saccades, covert saccades can also be detected during a quantitative video HIT, which would be undetectable in bedside examinations^36^. In this study, 14 patients showed overt saccades during video HITs (42.4%) and approximately half of the patients had concomitant covert saccades (8/14, 57.1%). However, none of the patients showed only covert saccades alone.

Catch-up saccades were predominantly observed in SCA3 (83.3%) and SCA6 (80%) patients, but were rarely observed in SCA7 (11.1%) and were absent in SCA2 patients. A previous comparative study regarding vestibular catch-up saccades in patients with slow visual saccades (4 of 12 enrolled patients were SCA2), vestibular disorders, and normal control concluded that overt saccades during video HITs in patients with slow saccades and normal control might resemble their visually guided saccades^37^. The study showed that patients with slow visual saccades showed normal mean VOR gains but also showed the lowest acceleration of overt saccades on the video HIT and a reduction in visual saccade velocity^37^. Moreover, SCA2 patients showed slower mean peak velocities of overt saccades than other groups^8,37^. These results indicated that catch-up saccades might be affected by the function of the saccadic generator^37^. Although the scant catch-up saccades in SCA2 and SCA7 might be caused by spared VOR gains for the HCs, saccadic generator dysfunction might also contribute to the paucity of catch-up saccades as well as scant GEN, considering that horizontal saccadic slowing is a cardinal ocular motor sign in SCA2 and SCA7, as replicated by our study. In this perspective, SCA6 patients without saccadic slowing showed markedly decreased VOR gains for the PCs and were frequently accompanied by catch-up saccades in the PCs. By contrast, in the SCA7 patients, catch-up saccades in the vertical canals were absent even in patients with decreased VOR gains for both vertical canals.

From this study, four patients with catch-up saccades in the HCs showed normal VOR gains for the HCs. Catch-up saccades are often documented without decrease of the VOR gains in conditions such as: (1) normal subjects with aging, (2) recovery phase after acute vestibular loss, and (3) various neurological diseases (i.e., vestibular migraine, SCA6, Friedrich’s ataxia, and multiple sclerosis), although the mechanisms and clinical significance remains uncertain^38,39^. Various patterns of saccadic movements have been observed during video HIT in central vestibular disorders associated with pre-existing ocular motor disturbances including nystagmus, ophthalmoplegia as well as the structural involvement^38^. Moreover, SN or GEN can interfere with VOR recordings and appear as ocular motor artifacts mimicking true refixation catch-up saccades^39^.

### Correlation of HITs with clinical parameters, and ocular motor characteristics

Disease severity assessed by the SARA showed a negative correlation with the VOR gains for the HCs in the SCA3 patients. Several studies have also reported a negative correlation between disease severity and VOR gains in SCA patients, which suggests that a reduction in VOR gains might reflect disease progression in SCA^6,8,12^. Even though the methods to evaluate clinical deterioration and the VOR gains varied between studies, the inverse correlation might support the clinical value of VOR assessment in SCA patients.

Ocular motor abnormalities are common in the SCAs even on initial assessment, and well-correlated with disease duration and severity^40^. The ocular motor characteristics of this study were mostly consistent with the previous studies^2,25,31,40–42^. Paucity of GEN and saccadic slowing were common in the SCA2 and SCA7 patients, whereas spontaneous and positional nystagmus (including perverted HSN) was frequently observed in the SCA6 patients, which were all known to be distinctive ocular motor features for each type of SCA^2,42^. Smooth pursuit abnormalities and saccadic dysmetria were common in all SCA patients regardless of subtype, which can be observed in diffuse cerebellar diseases involving the dorsal vermis/caudal fastigial nucleus or flocculus/paraflocculus^31,42^.

### Current and future directions for the pharmacological management of SCA

In general, therapeutic approaches for SCA are very difficult due to the rarity of the disease and its heterogeneity according to the genotypes^43^. Current trials mostly focus on ataxia rather than other symptoms such as VOR dysfunction^43^. Although several pharmacological treatment options including riluzole (activates calcium-dependent potassium channels, causing inhibition of deep cerebellar nuclei and decreasing cerebellar hyperexcitability), valproic acid (neuroprotective properties as a pan-histone deacetylase inhibitor), varenicline (modulate the activity of both Purkinje cells and granule cells), and lithium carbonate (anti-depressive properties) showed encouraging results from randomized clinical trials, there are still no disease-modifying treatments for SCAs^43–45^. Also, recent studies have raised the potential effects of nilotinib, acetyl-DL-leucine, and coenzyme Q10 on SCA^46–48^. However, clinical trials related to these drugs are in the early stages, and more evidence is needed to obtain conclusive results^46–48^.

As introduced in our study, VOR impairments are also common in patients with SCA, and they may be associated with additional vestibular symptoms and signs, such as oscillopsia, dizziness/vertigo and central types of nystagmus^5^. Indeed, several pharmacological agents including 3,4-diaminopyridine and 4-aminopyridine showed effect on downbeat nystagmus, dizziness, and oscillopsia in patients with SCA, even they failed to improve ataxic symptoms^49,50^. Taken together these observations and our study, VOR function in SCAs could be a potential indicator for treatment efficacy or a treatment target itself.

### Strengths and limitations

The primary strength of this study is that it used a valid, quantitative, and identical method to evaluate vestibular performance in the various subtypes of SCA. There are only a few studies that have analyzed the VOR in the vertical plane, which could be particularly important in cerebellar disease^6,12,29^. Another strength is that the VOR of SCA7 patients, which has also only been occasionally studied, was comparatively analyzed. A distinctive pattern of VOR impairments in SCA7 from this study is required to be further verified, but is expected to provide a better understanding of its neuropathology and to be a clinically differentiating factor from the other cerebellar ataxias.

This study also has limitations. For example, the study’s statistical power might be restricted because of the small number of patients in each subtype. However, there were similar patterns of VOR injury among the subjects, which correlated relatively closely with previous studies^7,8,13,31^. Further studies with larger sample sizes are essential to confirm and extend these results. Also, our study is a cross-sectional case–control study. Since VOR parameters including gains as well as ocular motor findings may change at the more advanced stage of each subtype, a follow-up cohort study may be needed to solve this problem.

## Conclusion

VOR impairments are common in cases of SCA. However, their patterns vary depending on the subtype. The VOR gains tended to be spared in the SCA2 subgroup but decreased markedly for all six semicircular canals in the SCA3 subgroup. In patients with SCA6, the VOR gains were significantly decreased for the PCs while the VOR gains for the ACs were relatively spared. The VOR gains in the SCA7 subgroup were decreased for the vertical canals (both ACs and PCs). The AC/PC ratio of the VOR gains was increased in SCA6 but decreased in SCA7. In addition to the ocular motor characteristics assessed by VOG, distinctive patterns of vestibular performance for each subtype using video HITs may aid the differential diagnosis of SCA genotypes. Also, beyond the cost-effectiveness, it may lead to a better understanding of the neuropathology specific to each subtype of SCA. Therefore, VOR assessment in conjunction with detailed neuro-otologic, and neuro-ophthalmologic evaluations are warranted for the patients presenting with cerebellar ataxia.

### Supplementary Information

Supplementary Legends.

Supplementary Figure S1.

Supplementary Figure S2.

Supplementary Tables.

## Data Availability

The datasets used and/or analysed during the current study available from the corresponding author on reasonable request.
