# Supplementary material for: Clinical value of vestibulo-ocular reflex in the differentiation of spinocerebellar ataxias
Source: Sci Rep. 2023 Sep 7;13:14783. doi: 10.1038/s41598-023-41924-6 (PMC10485070; doi:10.1038/s41598-023-41924-6)
Supplement: Supplementary file 1 — Supplementary Legends. [file 41598_2023_41924_MOESM1_ESM.docx]

**Supplementary Figure legends**

**Supplementary Fig. S1. Brain magnetic resonance images of patients with spinocerebellar ataxia**

Axial T1-weighted or fluid-attenuated inversion recovery (FLAIR) images of the patients indicate cerebellar atrophy with or without brainstem atrophy.

^*^Denotes the cases combined with brainstem atrophy

**Supplementary Fig. S2. Results of the video head impulse test: representative cases for each subtype of spinocerebellar ataxia (SCA)**

(A) In a patient with SCA2 (**Patient 8**), the vestibular-ocular reflex (VOR) gains for all semicircular canals are spared. (B) In SCA3 (**Patient 12**), the VOR gains are generally decreased, and abnormal catch-up saccades (both overt and covert saccades) are documented in both horizontal canal planes with right-sided predominance. (C) In SCA6 (**Patient 16**), the VOR gains for the posterior canals are significantly decreased and abnormal catch-up saccades can be seen in both posterior canal planes. (D) In SCA7 (**Patient 27**), the VOR gains for the vertical canals (both anterior and posterior canals) are decreased.

Abbreviations: AC, anterior canal; HC, horizontal canal; PC, posterior canal; Rt., right; Lt., left. Figures marked in red indicate decreased VOR gains, defined as <0.8 for the horizontal canal, and <0.73 for the vertical canals.
