# Supplementary figures and images for: Clinical value of vestibulo-ocular reflex in the differentiation of spinocerebellar ataxias
Source: Sci Rep. 2023 Sep 7;13:14783. doi: 10.1038/s41598-023-41924-6 (PMC10485070; doi:10.1038/s41598-023-41924-6)

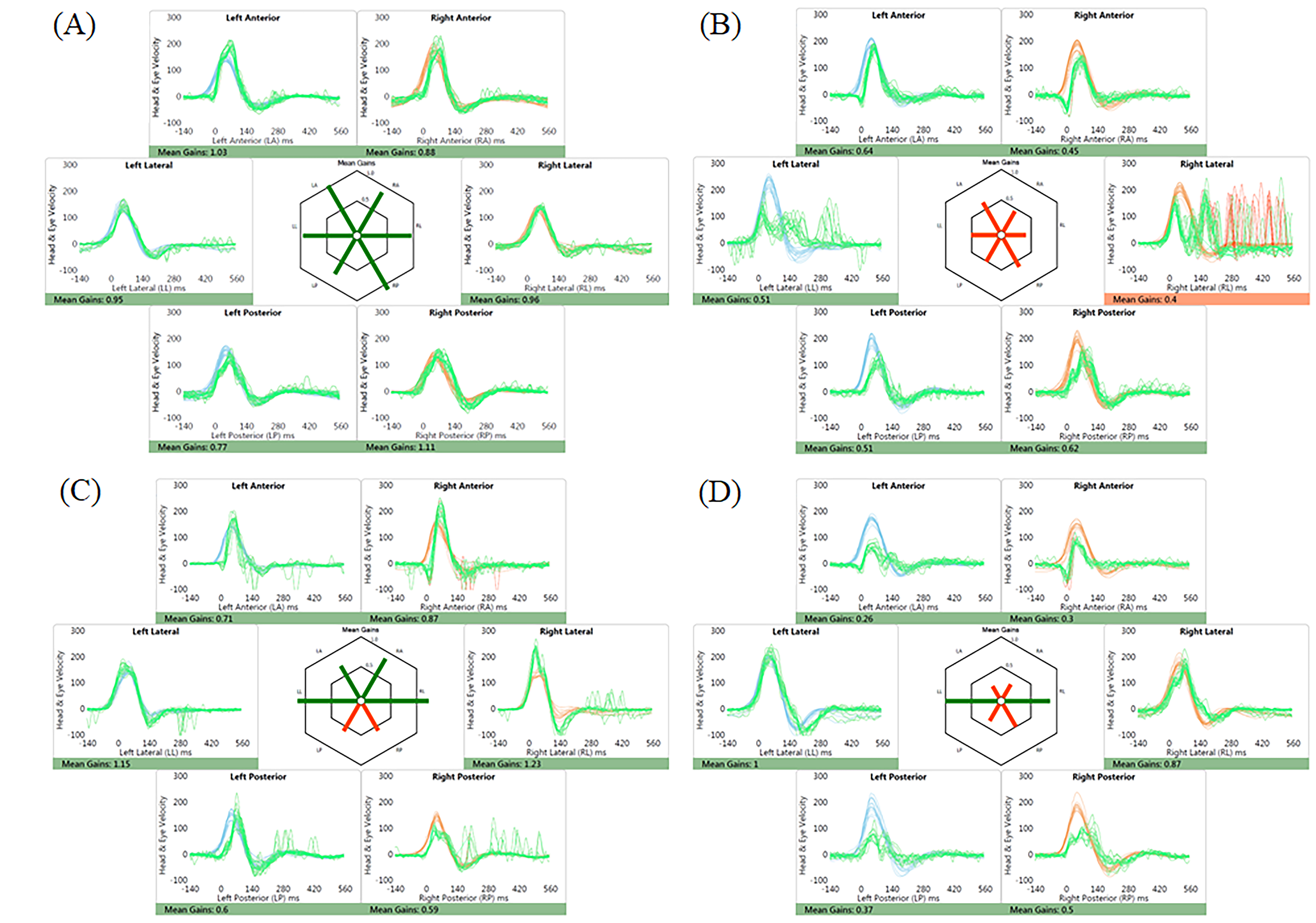

Supplement: Supplementary file 3 — Supplementary Figure S2. [file 41598_2023_41924_MOESM3_ESM.tif]
