# Supplementary material for: Clinical value of vestibulo-ocular reflex in the differentiation of spinocerebellar ataxias
Source: Sci Rep. 2023 Sep 7;13:14783. doi: 10.1038/s41598-023-41924-6 (PMC10485070; doi:10.1038/s41598-023-41924-6)
Supplement: Supplementary file 4 — Supplementary Tables. [file 41598_2023_41924_MOESM4_ESM.docx]

**Supplementary Table S1. Baseline characteristics and detailed clinical information of each patient**

| Pt. No. | Sex | Diagnosis | Age at onset | Age at diagnosis | Disease duration (yr)* | CAG repeat length | SARA | Initial neurologic symptoms | Other associated neurologic symptoms |
| --- | --- | --- | --- | --- | --- | --- | --- | --- | --- |
| 1 | F | SCA2 | 19 | 19 | 2 | 22/45 | N/A | Intermittent imbalance | Tremor |
| 2 | M | SCA2 | 26 | 27 | 1 | 22/43 | 12.0 | Intermittent imbalance |  |
| 3 | F | SCA2 | 34 | 38 | 8 | 22/41 | 12.0 | Intermittent imbalance |  |
| 4 | M | SCA2 | 20 | 34 | 15 | 22/41 | 14.0 | Gait ataxia | Muscle cramping, dystonia |
| 5 | M | SCA2 | 34 | 35 | 16 | 23/44 | 17.0 | Gait ataxia, dysarthria | Muscle cramping, dystonia |
| 6 | M | SCA2 | 31 | 32 | 1 | 22/40 | 2.0 | Intermittent imbalance |  |
| 7 | F | SCA2 | 61 | 64 | 3 | 22/37 | 13.5 | Intermittent imbalance | Tremor |
| 8 | M | SCA2 | 26 | 29 | 3 | 22/44 | 8.5 | Intermittent imbalance | Tremor |
| 9 | F | SCA3 | 51 | 53 | 2 | 27/71 | 12.0 | Gait ataxia, dysarthria | Muscle cramping, sensory neuropathy |
| 10 | M | SCA3 | 42 | 52 | 10 | 14/73 | 13.0 | Dizziness | Parkinsonism |
| 11 | M | SCA3 | 21 | 21 | 7 | 20/79 | N/A | Gait ataxia | Spasticity, dystonia |
| 12 | M | SCA3 | 34 | 43 | 14 | 15/70 | 22.0 | Gait ataxia | Sensory neuropathy, tremor, bradykinesia |
| 13 | F | SCA3 | 55 | 57 | 2 | 14/66 | 15.0 | Gait ataxia |  |
| 14 | F | SCA3 | 54 | 58 | 4 | 14/67 | 10.0 | Intermittent imbalance | Muscle cramping, dystonia |
| 15 | F | SCA6 | 39 | 42 | 3 | 13/26 | 11.0 | Dizziness |  |
| 16 | M | SCA6 | 35 | 35 | <1 | 13/27 | 21.0 | Dizziness |  |
| 17 | M | SCA6 | 32 | 34 | 8 | 13/26 | N/A | Gait ataxia, dysarthria |  |
| 18 | M | SCA6 | 35 | 38 | 14 | 13/27 | N/A | Gait ataxia |  |
| 19 | F | SCA6 | 39 | 42 | 12 | 13/27 | N/A | Gait ataxia |  |
| 20 | F | SCA6 | 44 | 45 | 11 | 13/26 | 26.0 | Gait ataxia |  |
| 21 | M | SCA6 | 50 | 53 | 3 | 12/23 | 3.0 | Dysarthria, Intermittent imbalance |  |
| 22 | F | SCA6 | 30 | 37 | 29 | N/A | 34.0 | Dizziness, intermittent imbalance |  |
| 23 | F | SCA6 | 53 | 56 | 11 | 16/21 | 12.0 | Dysarthria, tremor |  |
| 24 | M | SCA6 | 54 | 57 | 13 | 11/23 | 21.0 | Dizziness, gait ataxia |  |
| 25 | F | SCA7 | 12 | 15 | 5 | 10/57 | N/A | Intermittent imbalance, tremor | Retinal pigmentation, cone dystrophy, visual loss |
| 26 | F | SCA7 | 15 | 18 | 3 | 11/53 | 14.0 | Dizziness, intermittent imbalance | Retinal pigmentation, visual loss |
| 27 | F | SCA7 | 18 | 19 | 6 | 9/50 | 25.0 | Gait ataxia | Retinal pigmentation, cone dystrophy, visual loss |
| 28 | M | SCA7 | 34 | 35 | 7 | 11/42 | N/A | Gait ataxia, dysarthria | Retinal pigmentation, cone dystrophy, visual loss |
| 29 | F | SCA7 | 26 | 34 | 8 | 10/45 | 23.0 | Bradykinesia, dysarthria | Retinal degeneration, visual loss |
| 30 | M | SCA7 | 47 | 49 | 2 | 10/40 | 13.0 | Intermittent imbalance | Retinal degeneration, visual loss |
| 31 | M | SCA7 | 54 | 55 | 2 | 10/40 | 7.5 | Gait ataxia | Retinal degeneration, visual loss |
| 32 | M | SCA7 | 41 | 45 | 10 | 10/41 | 13.0 | Bradykinesia | Retinal degeneration, visual loss |
| 33 | F | SCA7 | 22 | 22 | <1 | 10/54 | 8.0 | Gait ataxia | Retinal degeneration, visual loss |

*Disease duration was calculated from the age at onset to the age when the video head impulse test was performed. Pt.=patient, No.=number, M=male, F=female, SCA=spinocerebellar ataxia.

**Supplementary Table S2. Detailed information regarding the results of video head impulse test and ocular motor characteristics of each patient**

| Pt. No. | Diagnosis | VOR gain | | | | | | | Overt saccades | Covert saccades | Bedside HIT | SN | GEN | Saccades | Smooth pursuit | HSN | PN |
| --- | --- | --- | --- | --- | --- | --- | --- | --- | --- | --- | --- | --- | --- | --- | --- | --- | --- |
|  |  | RA | RH | RP | LA | LH | LP | AC/PC |  |  |  |  |  |  |  |  |  |
| 1 | SCA2 | 0.88 | **1.36** | 1.06 | 0.98 | 1.14 | 0.98 | 0.91 | - | - | - | - | - | Slowing, hypometria | Decreased gain, SP | - | - |
| 2 | SCA2 | **1.14** | **1.42** | 1.03 | 0.86 | **1.44** | **1.17** | 0.91 | - | - | - | - | - | Slowing | Decreased gain, SP | - | - |
| 3 | SCA2 | 1 | 1.15 | 0.81 | 0.75 | 1.01 | 0.74 | 1.13 | - | - | - | - | - | Slowing | Normal gain, SP | - | - |
| 4 | SCA2 | 1.01 | 1.02 | 0.8 | 0.93 | 1.18 | 0.74 | 1.26 | - | - | - | - | - | Slowing | Decreased gain, SP | - | - |
| 5 | SCA2 | 1.07 | 1.11 | **0.64** | **0.71** | 0.95 | 0.8 | 1.24 | - | - | - | - | - | Slowing, hypometria | Decreased gain, SP | - | - |
| 6 | SCA2 | 0.97 | 1.02 | 0.84 | 0.74 | 0.89 | 0.87 | 1 | - | - | - | SWJ | - | Spared | Normal gain, SP | - | - |
| 7 | SCA2 | 0.86 | 0.94 | **1.2** | **1.14** | 0.91 | **1.17** | 0.84 | - | - | - | - | - | Slowing, hypometria | Decreased gain, SP | - | - |
| 8 | SCA2 | 0.88 | 0.96 | **1.11** | 1.03 | 0.95 | 0.77 | 1.02 | - | - | - | - | - | Slowing, hypometria | Normal gain, SP | - | - |
| 9 | SCA3 | **0.47** | **0.6** | **0.68** | **0.67** | 0.86 | **0.45** | 1.01 | LH 1+, RH 2+ | RH 1+ | Both | MSO | + | Hypermetria | Decreased gain, SP | - | - |
| 10 | SCA3 | **0.41** | **0.66** | **0.59** | **0.48** | **0.51** | **0.43** | 0.87 | LH 1+ | - | Lt.† | - | - | Hypermetria | Decreased gain, SP | - | - |
| 11 | SCA3 | 0.76 | **0.71** | **0.62** | **0.72** | **0.44** | **0.59** | 1.22 | - | - | - | - | + | Slowing, hypometria | Decreased gain, SP | - | - |
| 12 | SCA3 | **0.45** | **0.4** | **0.62** | **0.64** | **0.51** | **0.51** | 0.97 | LH 1+, RH 2+ | LH 1+, RH 2+ | Rt. | - | + | Slowing | Decreased gain, SP | - | - |
| 13 | SCA3 | **0.71** | **0.69** | 0.86 | **0.67** | **0.67** | 0.73 | 0.87 | LH 2+, RH 2+ | LH 2+ | Both | SWJ | + | Hypometria | Decreased gain, SP | - | - |
| 14 | SCA3 | **0.61** | 0.84 | 1.07 | **0.7** | 0.91 | 0.71 | 0.74 | RH 2+ | LH 1+ | - | - | + | Hypometria | Decreased gain, SP | - | - |
| 15 | SCA6 | 0.78 | **0.74** | **0.67** | **0.72** | **0.74** | **0.6** | 1.18 | RP 1+ | RP 1+ | Lt. † | DBN | + | Spared | Decreased gain, SP | DBN | Apo |
| 16 | SCA6 | 0.87 | 1.23 | **0.59** | **0.71** | 1.15 | **0.6** | 1.33 | LP 1+. RP 1+ | RP 1+ | - | LBN, DBN | + | Hypometria | Decreased gain, SP | LBN | Apo |
| 17 | SCA6 | 0.94 | 1.05 | 0.8 | 0.9 | 1.04 | 0.84 | 1.12 | - | - | - | DBN | + | Hypometria | Normal gain, SP | DBN | - |
| 18 | SCA6 | 0.96 | 0.81 | 0.89 | 1.13 | 0.9 | 0.85 | 1.20 | LH 1+ | - | Both | RBN | + | Hypometria | Decreased gain, SP | - | Apo |
| 19 | SCA6 | 0.8 | **0.63** | **0.55** | 0.73 | **0.7** | **0.47** | 1.5 | RP 1+ | - | Both | RBN | +,＊ | Hypermetria | Normal gain, SP | RBN, DBN† | Geo |
| 20 | SCA6 | 1.07 | 1.02 | **0.66** | 0.92 | **0.77** | **0.62** | 1.56 | LH 1+, RH 2+ | - | Lt. | LBN | +,＊ | Hypermetria | Normal gain, SP | RBN🡺LBN, DBN† | Apo |
| 21 | SCA6 | 0.99 | **1.35** | 1.07 | 0.89 | 1.15 | 0.92 | 0.95 | - | - | - | - | - | Spared | Decreased gain, SP | DBN† | Apo |
| 22 | SCA6 | 0.92 | 0.99 | **0.71** | 0.9 | **0.57** | 0.85 | 1.17 | LH 2+ | - | Lt. | - | + | Hypometria | Decreased gain, SP | DBN† | - |
| 23 | SCA6 | 0.79 | 1.11 | 0.76 | 0.86 | 0.89 | **0.68** | 1.15 | LH 1+, RH 1+ | LH 1+ | Both† | - | + | Hypometria | Decreased gain, SP | RBN | - |
| 24 | SCA6 | 0.6 | 1.31 | 0.31 | 0.69 | 1.23 | **0.39** | 1.84 | LH 2+, RP 1+, LP 1+ | LH 1+ | Lt.† | DBN | + | Hypometria | Normal gain, SP | - | Apo |
| 25 | SCA7 | **0.47** | **0.75** | **0.47** | **0.33** | **0.68** | **0.63** | 0.73 | - | - | - | - | - | Slowing | Normal gain, SP | - | - |
| 26 | SCA7 | 0.76 | 0.97 | 1.03 | 0.85 | 1.05 | 0.78 | 0.89 | - | - | - | - | - | Slowing, hypometria | Decreased gain, SP | - | - |
| 27 | SCA7 | **0.3** | 0.87 | **0.5** | **0.26** | 1 | **0.37** | 0.64 | - | - | Lt. | - | - | Slowing | Decreased gain, SP | - | - |
| 28 | SCA7 | **0.62** | **0.59** | 0.86 | **0.62** | **0.69** | **0.71** | 0.79 | - | - | - | - | - | Hypermetria | Normal gain, SP | - | - |
| 29 | SCA7 | **0.64** | 1.21 | **0.65** | **0.43** | 1.01 | **0.6** | 0.86 | - | - | - | - | - | Slowing, hypometria | Decreased gain, SP | - | - |
| 30 | SCA7 | **0.65** | **0.57** | 0.76 | **0.41** | 0.9 | 0.8 | 0.68 | RH 2+ | - | Both | - | - | Slowing | Normal gain, SP | - | - |
| 31 | SCA7 | 0.74 | **1.39** | 0.88 | 0.76 | 1.2 | 0.91 | 0.84 | - | - | - | - | - | Slowing, hypermetria | Decreased gain, SP | - | - |
| 32 | SCA7 | **0.55** | 0.96 | 0.76 | **0.39** | 0.92 | 0.76 | 0.62 | - | - | - | - | - | Slowing, hypermetria | Normal gain, SP | - | - |
| 33 | SCA7 | **0.7** | 0.92 | 0.94 | **0.72** | 0.96 | 0.8 | 0.82 | - | - | - | - | - | Slowing, hypometria | Normal gain, SP | - | - |

＊centripetal nystagmus, †perverted, overt & covert saccades were categorized according to the frequency of catch-up saccades; -=none, 1+=less than 50%, 2+=more than 50%. Figures marked in bold indicate abnormal VOR gain which was defined when the estimated gain value deviated from the mean ± 2SD obtained from the control group
Pt.=patient, No.=number, VOR=vestibulo-ocular reflex, HC=horizontal canal, AC=anterior canal, PC=posterior canal, HIT=head impulse test, SN=spontaneous nystagmus, GEN=gaze-evoked nystagmus, HSN=headshaking-induced nystagmus, PN=positional nystagmus, SCA=spinocerebellar ataxia, SP=saccadic pursuit, LH=left horizontal canal, RH=right horizontal canal;, RP=right posterior canal, LP=left posterior canal, RA=right anterior canal, SWJ=square-wave jerk, MSO=macrosaccadic oscillation, LBN=left-beating nystagmus, RBN=right-beating nystagmus, DBN=downbeat nystagmus, Apo=apogeotropic nystagmus in the supine head roll test, Geo=geotropic nystagmus in the supine head roll test.

**Supplementary Table S3. Correlations between the vestibulo-ocular reflex gain and clinical parameters**

| Total (n=33) | HC gain | AC gain | PC gain | AC/PC ratio | SARA^‡^ | CAG repeat length |
| --- | --- | --- | --- | --- | --- | --- |
| **SCA2 (n=8)** |  |  |  |  |  |  |
| CAG repeat length | 0.446 | -0.073 | -0.157 | 0.193 | 0.156 | 1 |
| Age at onset | -0.639 | -0.115 | 0.000 | -0.145 | 0.165 | -0.579 |
| SARA | 0.406 | 0.373 | -0.450 | 0.450 | 1 | 0.156 |
| Disease duration^†^ | -0.169 | -0.073 | -0.699 | 0.747^*^ | 0.798^*^ | 0.091 |
| **SCA3 (n=6)** |  |  |  |  |  |  |
| CAG repeat length | -0.429 | -0.086 | -0.580 | 0.771 | 0.441 | 1 |
| Age at onset | 0.771 | 0.086 | 0.493 | -0.771 | -0.821 | -0.829^*^ |
| SARA | -0.900* | -0.200 | -0.359 | 0.300 | 1 | -0.100 |
| Disease duration | -0.754 | -0.493 | -0.485 | 0.174 | 0.410 | 0.406 |
| **SCA6 (n=10)** |  |  |  |  |  |  |
| CAG repeat length (n=9) | -0.511 | 0.139 | -0.173 | 0.295 | 0.297 | 1 |
| Age at onset | 0.419 | -0.298 | -0.298 | 0.231 | -0.264 | -0.783^*^ |
| SARA | -0.216 | 0.180 | -0.162 | 0.505 | 1 | 0.373 |
| Disease duration | -0.304 | 0.103 | 0.018 | 0.249 | 0.545 | 0.013 |
| **SCA7 (n=9)** |  |  |  |  |  |  |
| CAG repeat length | -0.193 | -0.100 | -0.192 | 0.201 | 0.035 | 1 |
| Age at onset | 0.243 | 0.200 | 0.283 | -0.150 | -0.199 | -0.946^**^ |
| SARA | -0.264 | -0.450 | -0.631 | 0.000 | 1 | 0.513 |
| Disease duration | -0.118 | -0.452 | -0.527 | -0.301 | 0.426 | 0.609 |

^*^p<0.05, ^**^p<0.01, ^†^Disease duration was calculated from the age at onset to the age when the video head impulse test was performed. ^‡^SARA was checked in 26 patients (SCA2=7, SCA3=5, SCA6=7, SCA7=7). HC=horizontal canal, AC=anterior canal, PC=posterior canal, SCA=spinocerebellar ataxia, SARA=Scale for the Assessment and Rating of Ataxia
